# Dementia caregiver burgen in a brasilian sample: Association to neuropsychiatric symptoms

**DOI:** 10.1590/S1980-57642009DN30200011

**Published:** 2009

**Authors:** Patrícia Paes Araujo Fialho, Anne Marise Koenig, Etelvina Lucas dos Santos, Henrique Cerqueira Guimarães, Rogério Gomes Beato, Viviane Amaral Carvalho, Thais Helena Machado, Paulo Caramelli

**Affiliations:** 1 MSc, Behavioral and Cognitive Neurology Research Group, Department of Internal Medicine, Faculty of Medicine, Federal University of Minas Gerais, Belo Horizonte, MG, Brazil.; 2 BSc, Behavioral and Cognitive Neurology Research Group, Department of Internal Medicine, Faculty of Medicine, Federal University of Minas Gerais, Belo Horizonte, MG, Brazil.; 3 MD, Behavioral and Cognitive Neurology Research Group, Department of Internal Medicine, Faculty of Medicine, Federal University of Minas Gerais, Belo Horizonte, MG, Brazil.; 4 MD, PhD, Behavioral and Cognitive Neurology Research Group, Department of Internal Medicine, Faculty of Medicine, Federal University of Minas Gerais, Belo Horizonte, MG, Brazil.

**Keywords:** dementia, neuropsychiatric symptoms, caregiver burden

## Abstract

Taking care of elderly demented individuals, especially when they present behavioral changes, can be very exhaustive for both family and caregivers. Generally, this leads to changes in the family lifestyle, and the caregiver must deal with a range of problems. Information on this topic in Latin America, including Brazil, remains scarce.

**Objective:**

To investigate the relationship between the presence of neuropsychiatric symptoms and the level of caregiver burden in a group of Brazilian elderly with dementia.

**Methods:**

The Brazilian versions of the Zarit Caregiver Burden Interview (ZBI) and of the Neuropsychiatric Inventory (NPI) were administered to a total of 83 family-caregivers of patients with dementia followed at a university-affiliated outpatient clinic. Pearson’s correlations were calculated to measure the level of association between the scores on both instruments.

**Results:**

Among the caregivers, 83.1% were women, and had a mean age of 55.6±12.8 years. The ZBI scores ranged from 3 to 79 (mean=31.4). Patients’ NPI scores ranged from 0 to 102 (mean=26.9), consistent with a significant degree of behavioral manifestations in most patients. A significant positive correlation was found between ZBI and NPI scores (r=0.402; p=0.000).

**Conclusion:**

The presence and severity of behavioral manifestations assessed by the NPI were associated with a high level of caregiver burden in this sample of Brazilian elderly with dementia.

## Abstract (pt)

Cuidar de idosos com demência, especialmente se eles apresentam transtornos de comportamento, pode ser extremantente exaustivo para os familiares e cuidadores. Geralmente, ocorrem mudanças no estilo de vida da família, e o cuidador precisa lidar com grande diversidade de problemas. Informações sobre este tema na América Latina, incluindo o Brasil, ainda permanece escasso.

**Objetivo:**

Investigar a relação entre a presença de sintomas neuropsiquiátricos e o nível de sobrecarga do cuidador em um grupo de idosos brasileiros com demência.

**Métodos:**

As versões brasileiras do Inventário de Sobrecarga de Zarit (ISZ) e do Inventário Neuropsiquiátrico (INP) foram administrados a um total de 83 cuidadores-familiares de pacientes com demência acompanhados em um ambulatório universitário. A correlação de Pearson foi calculada para medir o nível de associação entre os escores em ambos os instrumentos.

**Resultados:**

Entre os cuidadores, 83,1% eram mulheres, com idade média de 55,6±12,8 anos. Os escores do ISZ variaram entre 3 a 79 (média=31,4). Os escores do INP dos pacientes alcaçaram escores entre 0 a 102 (média=26,9), compatível com elevado grau de manifestações comportamentais na maioria dos pacientes. Uma correlação positiva significativa foi encontrada entre os escores obtidos no ISZ e o INP (r=0,402; p=0,000).

**Conclusão:**

A presença e a gravidade das manifestações comportamentais avaliadas pelo INP estão associadas ao alto nível de sobrecarga do cuidador nesta amostra de idosos brasileiros com demência.

The care of patients with dementia is usually provided by the family, particularly by women (spouse, daughter, granddaughter or sister).^1-4^ Apart from the cognitive and functional changes which occur in dementia, family-caregivers commonly have to deal with neuropsychiatric disturbances, such as apathy, depression, psychotic symptoms, agitation and aggression. These clinical manifestations increase over time and, depending on the their severity, require considerable changes in the family structure.^1^

Very often the caregiver is inexperienced and unprepared to deal with the behavioral and personality changes of the patient. Adverse effects on the mental health of caregivers have been reported and some studies have demonstrated the benefits of interventions directed to caregivers in the form of support programs.^2,5-10^ Taking care of elderly demented people involves complex and specific procedures which can be imparted to the family-caregiver by specialists according to each case, in an individualized approach. Therefore, a necessary prerequisite to devising and evaluating interventions focused on the caregiver of dementia patients is to investigate the presence and severity of neuropsychiatric disturbances as well as the level of caregiver burden.

The Neuropsychiatric Inventory (NPI) was developed for the assessment of a wide range of behaviors encountered in dementia patients and provides means of evaluating frequency and severity of behavioral changes. Different behavioral domains are covered by the NPI, namely, delusions, hallucinations, dysphoria, anxiety, agitation/aggression, euphoria, disinhibition, irritability/lability, apathy, aberrant motor activity, night-time behavior disturbances and appetite/eating abnormalities.^11^ The Zarit Caregiver Burden Interview (ZBI) was developed to evaluate the caregiver’s health condition, psychological well-being, finances, and social life.^1^

The objective of the present study was to examine the relationship between the presence of neuropsychiatric changes in a group of elderly patients with dementia and the level of caregiver burden, by means of the two instruments mentioned above, the NPI and the ZBI.

## Methods

A convenient sample comprising 83 family-caregivers of patients followed at the Behavioral and Cognitive Neurology Outpatient Clinic of the Hospital das Clínicas of the Federal University of Minas Gerais (BCNOC-HCUFMG), in Belo Horizonte, Brazil and family-caregivers of demented elderly living in the city of Caeté, Minas Gerais state, in Brazil, who agreed to participate in the study were included in this exploratory survey.

The caregivers had to have at least 12 hours of patient contact time per week, in order to be included in the study. All patients were examined by physicians of the BCNOC-HCUFMG research group. The patients had to fulfill the DSM-IV diagnostic criteria for dementia^12^ but the etiology and the severity of dementia were not taken into account.

A structured interview was administered to all caregivers, with questions related to sociodemographic characteristics and knowledge about dementia and on the caregiver role. As for the latter information, we asked if the caregiver had received any kind of information about caregiving in the past, irrespective of the source (medical staff, group discussion, educational material, etc.).

Frequency and severity of behavioral disorders were measured using the Brazilian version of the NPI,^13^ while caregiver burden was appraised by the Brazilian version of the ZBI.^1^ Pearson’s correlations were calculated to measure the level of association between the scores on both instruments. The scores on the ZBI were analyzed by the Mann-Whitney test according to gender and age, with age divided into two groups (Group 1: up to 55 years; Group 2: >55 years). The study was approved by the Ethics Committee from the Federal University of Minas Gerais and all caregivers who agreed to take part signed the Informed Consent Form.

## Results

Overall, 95 caregivers were recruited for the study, although we were able to fully interview and to administer the ZBI and the NPI to 83 of these. The caregivers were aged 28 to 92 years, 83.1% women, and had 1 to 16 years of schooling. In general, 40% of the family caregivers were married to the patients and 43% were either their son or daughter. About 39% of the sample had been in charge of the patient for at least five years. Forty-six percent of caregivers received help from other family members, while 12% had professional assistance. The majority (66%) of caregivers received no guidance on the role of caregiver. Table 1 depicts the sociodemographic and the caregiving profiles of the family caregivers.

**Table 1:** Sociodemographic profile and caregiving characteristics of family caregivers.

Total sample N=83	
Mean age (SD)	55.6 (12.8)
Gender	
Male	16.9% (n=14)
Female	83.1% (n=69)
Mean schooling (SD)	8.2 (4.4)
Familial relationship	
Spouse	39.8%
Children	43.4%
Other	16.9%
Caregiver for	
< 3 years	34.9%
3 to 5 years	26.5%
> 5 years	38.6%
Task distribution	
Single caregiver	42.2%
Family assistance	45.8%
Professional assistance	12.0%
Previous guidance	
Yes	33.7%
No	66.3%

The ZBI scores ranged from 3 to 79 (mean=31.4; SD=16.0), whereas patients’ NPI scores ranged from 0 to 102 (mean=26.9; SD=22.9). A positive correlation was found between ZBI and NPI scores (r=0.402; p<0.001). The gender and age subgroups did not present statistical difference with regard to burden level on the ZBI (p=0.484 and p=0.347, respectively) (Table 2).

**Table 2:** ZBI and INP score distributions.

	Caregivers										Patients
	Gender*				Age*				Total		Total
	Male n=14	Female n=69	p		£ 55 n=44	> 55 n=39	p		N=83		N=83
ZBI mean (SD)	27.71 (16.33)	32.09 (15.97)	0.484		29.84 (15.66)	33.05 (16.44)	0.347		31.35 (16.01)		N/A
INP mean (SD)	N/A	N/A			N/A	N/A			N/A		26.88 (22.86)

* Mann-Whitney test; N/A, not applicable.

## Discussion

This study analyzed the association between the level of caregiver burden and the presence of neuropsychiatric changes in a group of elderly patients with dementia. The level of burden was influenced by neuropsychiatric disturbances, as shown by the positive correlation found between ZBI and NPI scores. As expected, while patients’ NPI scores increased, the burden perceived by the caregivers also increased.

The interest concerning the well-being of caregivers of elderly patients is increasing in the literature. The more people age, the more common chronic illnesses become. The degree of burden that providing care to elderly demented people can cause depends largely on the clinical aspects of the patient. Moreover, the caregivers’ expectations and necessities also need to be taken into account. One of the most onerous aspects for the caregiver is coping with neuropsychiatric disturbances.^11,14,16,17^ Corroborating this point, Cassis et al.^16^ observed in a Brazilian setting that caregiver burden was associated with behavioral disorders, dependencies, cognitive impairment, and onset of symptoms, caregiving and co-residency. Among socio-demographics characteristics, lower levels of stress were found in black caregivers.

Neri and Carvalho^3^ commented that some variables such as gender and age are more strongly related to burden than others. They pointed out that young caregivers experience more burden than older ones and, similarly, that women experience more burden than men. In another study, females reported higher levels of depression and neuroticism than males.^7^ Torti et al.,^17^ in a multinational review, found that female caregivers bear a particularly heavy burden across cultures, particularly in Asian societies. However, and contrary to these previous studies, our results show that there are no significant differences in the burden level with regard to gender or age. These differences might be related to sample size and we expect to further explore these issues in future studies.

Another aspect of the present study concerns the level of burden in our sample. Family caregivers are often challenged to manage the long-term symptoms of dementia without adequate knowledge of the disease or support from other relatives or professionals.^18^ Our result showed that 66% of the caregivers reported that they had no guidance on the role of being a caregiver. Schreiner et al.^15^ suggest that a ZBI cut-off score from 24 to 26 would be useful in identifying caregivers at risk for depression and in need of further assessment and intervention. The mean score in our study was 31.4±16.0. These findings are indicative of a high level of burden in this sample of those who provide care to elderly patients with dementia. According to Truzzi et al.,^19^ in a Brazilian study, care burden was the most significant factor associated to burnout. Burnout is defined as extreme physical and mental fatigue, emotional exhaustion, decreased work motivation, and lack of empathy towards others.^18,20^ Moreover, burnout represents a problem that also occurs within the workplace. It is important to point out that not only the formal workplace, but also informal home care settings can induce burnout.^20^

Finally, while behavioral disorders are common and expected in the course of dementia, the lack of information on how caregivers should deal with these problems represents an additional weight to caregiver burden which is a crucial factor behind institutionalization of the patients.^11,21^ Torti et al.^17^ pointed out that interventions designed to reduce caregiver burden have been largely, although not universally, unsuccessful. They underline the failure of many traditional intervention programs coupled with the reported differences in caregiver coping across cultures. In this sense, there is a need to develop and test appropriate interventions tailored to meet the needs of the caregivers, combined with patients’ assistance.

The main focus of the present study was on the relationship between the presence of neuropsychiatric signs and the level of caregiver burden. Although the caregivers sample size was small, which naturally represents a limitation of our study, the findings suggest that the presence of behavioral changes is a possible indicator of a high level of caregiver burden. Providing relief to families could be just one important way to avoid institutionalization or perhaps to extend the stay of the elderly in the caregiver’s home besides reducing the stress and burden of caregivers.
